# Long-term survival in a patient with progressive multifocal leukoencephalopathy after therapy with rituximab, fludarabine and cyclophosphamide for chronic lymphocytic leukemia

**DOI:** 10.1186/s40164-015-0003-4

**Published:** 2015-03-01

**Authors:** Heidys Garrote, Adolfo de la Fuente, Raquel Oña, Inmaculada Rodríguez, Juan E Echevarría, Juan M Sepúlveda, Juan F García

**Affiliations:** Department of Translational Research, MD Anderson Cancer Center Madrid, Madrid, Spain; Department of Hematology, MD Anderson Cancer Center Madrid, Madrid, Spain; Department of Radiology, MD Anderson Cancer Center Madrid, Madrid, Spain; Department of Virology, Centro Nacional de Microbiología, Instituto de Salud Carlos III, Madrid, Spain; Centro de Investigación Biomédica en Red de Epidemiología y Salud Pública (CIBERESP), Madrid, Spain; Department of Neurology, MD Anderson Cancer Center Madrid, Madrid, Spain; Department of Pathology, MD Anderson Cancer Center, Madrid, Spain

**Keywords:** Progressive multifocal leukoencephalopathy, John Cunningham virus, Demyelization, Immune system suppression

## Abstract

A 50-year-old male with chronic lymphocytic leukemia (CLL) was treated with fludarabine, cyclophosphamide and rituximab, which produced a complete remission. Eight months after the last dose of rituximab he had visual disturbance, diminished muscular strength in the right arm and vesicular-papular lesions in the left ophthalmic branch region of the V cranial nerve. These were initially interpreted as herpes virus encephalopathy (HVE), but brain magnetic resonance imaging (MRI) showed evidence of demyelination consistent with progressive multifocal leukoencephalopathy (PML). Cerebrospinal fluid (CSF) analysis was negative for varicella zoster virus (VZV) and John Cunningham virus (JCV) DNA. The clinical suggestion of PML prompted us to perform a brain biopsy and to start treatment with mefloquine. In the brain biopsy, histopathological features of demyelination were described and the polymerase chain reaction (PCR) identified JCV, confirming the diagnosis of PML. Treatment with mefloquine (250 mg/week) and dexamethasone (4 mg/day) was started and maintained for 6 months. A year later there was an almost complete resolution of the MRI lesions and the patient achieved a stable clinical state with persisting motor impairment and severe epilepsy. The patient is alive 38 months after diagnosis of PML, which is the longest known survival to date.

## Background

Progressive multifocal leukoencephalopathy (PML) is a rare demyelinating condition of the central nervous system (CNS) that is frequently fatal. It is caused by reactivation of latent John Cunningham virus (JCV) in the context of immune system suppression, particularly in patients infected with human immunodeficiency virus (HIV) and in transplantation recipients, although it was first described in patients with chronic lymphocytic leukemia (CLL) and Hodgkin lymphoma [1,2]. Previous chemotherapy exposure and compromised immune systems are postulated risk factors.

The increasing number of PML cases recently diagnosed under monoclonal antibody therapy (rituximab, alemtuzumab, brentuximab and natalizumab) highlights the role of the immune system suppression in the pathogenesis of PML [3-6].

Rituximab is a CD20-specific monoclonal antibody that is effective in treating CLL. Rituximab therapy has been associated with reactivation of viral infections such as hepatitis B, cytomegalovirus, herpes simplex virus (HSV), varicella zoster virus (VZV), West Nile virus and JCV [3].

The pathophysiology of rituximab-associated PML is unclear; some findings suggest that hematopoietic progenitor cells may be a site of viral latency. Hematopoietic progenitor cells mobilized into the peripheral blood during chemotherapy may have been infected with latent JCV and may have facilitated the hematogenous spread of JCV into the central nervous system (CNS) [3].

The outcome of patients with PML is mostly unfavorable, leading to death in 90% with median survival of only two months [3]. Historically, treatment of PML has rarely been successful in the absence of immune system reconstitution. To date there are no reports in the literature of patients diagnosed with PML and survival beyond 25 months, although it is possible that longer-term survivors have not been reported.

## Case presentation

A 50-year-old Caucasian male patient presented with visual disturbance and diminished muscular strength in the right arm in August 2011.

He had a previous medical history of CLL in 2007 and had a stable clinical condition for the following three years, when increasing lymphocytosis, anemia, thrombocytopenia and splenomegaly occurred, indicating CLL in progression; he was then treated with six cycles of fludarabine, cyclophosphamide and rituximab, achieving complete remission (CR) by the end of the treatment in December 2010.

A neurological examination revealed subconjunctival erythema in the left eye and vesicular-papular lesions in the region of the left ophthalmic branch of the V cranial nerve, dysarthria and paresis of the right arm. He had no meningeal signs and no fever. There were no other medically relevant personal or family conditions.

The presence of the cutaneous lesions accompanying the neurological symptoms resulted in the condition being interpreted as herpes virus encephalopathy (HVE). Other entities such as vasculitis, lymphomatous meningiosis by the underlying CLL or stroke were ruled out by clinical and routine studies.

Treatment with intravenous acyclovir was initiated, which produced an improvement in the skin lesions but progressive deterioration of the neurological status of the patient.

Blood count measurements showed a hemoglobin level of 139 g/L, a total white cell count of 3.5×10^9^/L and a platelet count 98×10^9^/L. All biochemical parameters were normal.

Flow cytometry of the peripheral blood lymphocytes revealed a non-clonal cell population; CD4+ and CD8+ counts were 222 cells/μL (normal limits, 400–1300 cells/μL) and 405 cells/μL (normal limits, 200–700 cells/μL), respectively.

Serology showed the following results: negative for hepatitis B, hepatitis C, HIV and toxoplasmosis IgG and IgM. HSV type I IgG positive and IgM negative.

Cerebrospinal fluid (CSF) analysis indicated a normal cell count, and normal protein and glucose levels, with no evidence of neoplastic cells. Polymerase chain reaction (PCR) analysis of the CSF was negative for JCV, HSV RNA, cytomegalovirus RNA, herpes virus Type 6 (HVT6) DNA and VZV DNA.

**Figure 1:**
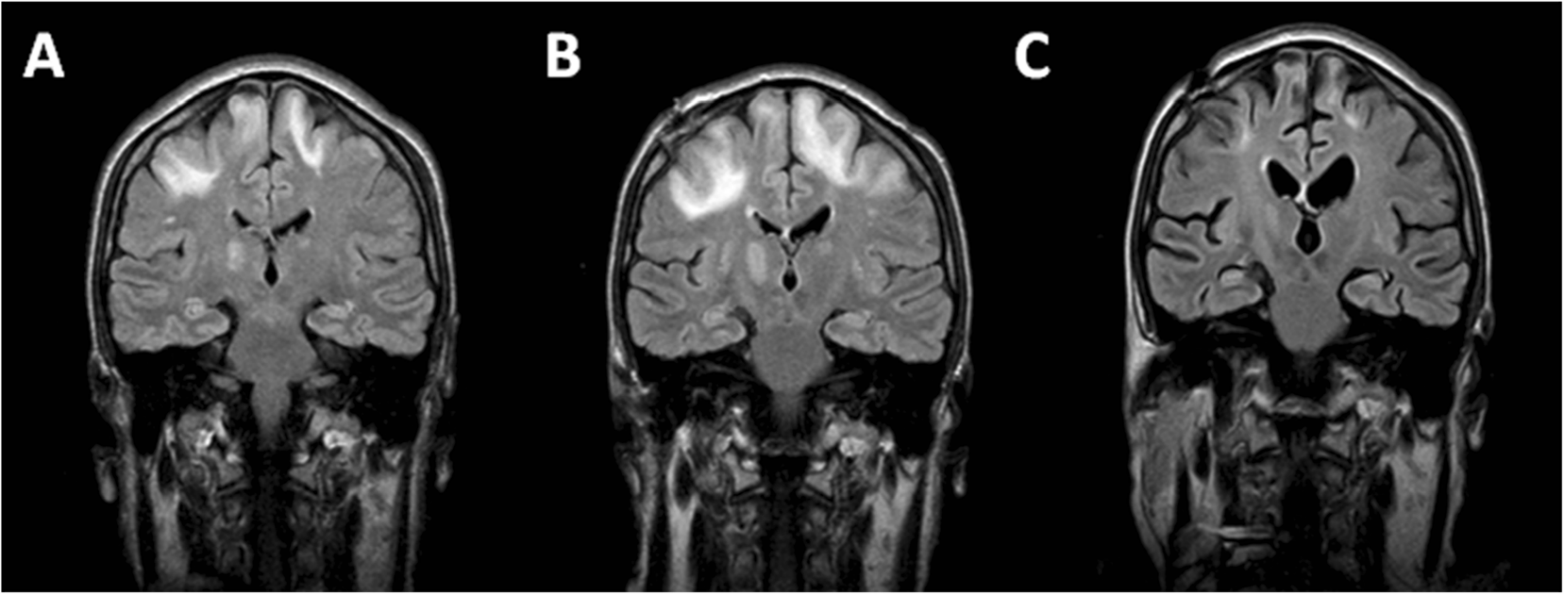
**Fluid-attenuated inversion recovery (FLAIR) images in the affected regions. A**: bilateral hyperintensity in the white matter involving the parietal and occipital lobules and the internal capsule. **B**: Increment in the extension and intensity of the parietal lesion, one month after diagnosis. **C**: Complete resolution of the lesion, one year after diagnosis.

Magnetic resonance imaging (MRI) of the brain showed a bilateral hyperintensity (Figure 1A) in the white matter involving the parietal and occipital lobules and the internal capsule; there was no mass effect, edema, hemorrhagic or ischemic lesions. Although the JCV was not detected in the CSF, the findings were consistent with demyelination and suggestive of PML and while waiting for the PCR results, empirical treatment was initiated with oral mefloquine and a brain biopsy was performed.

**Figure 2:**
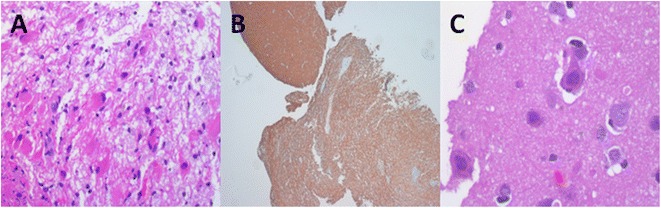
**Histological examination. A**: Infiltration of the brain tissue by foamy macrophages and mature lymphocytes with perivascular clustering. **B**: Immunohistochemical staining for neurofilaments revealed a loss of myelin. Compare affected tissue at the bottom with normal brain tissue at the upper corner of the picture. **C**: Reactive astrocytes with polymorphic nuclei and prominent nucleoli.

Histological examination of the sample revealed infiltration of the brain tissue by foamy macrophages and mature lymphocytes with perivascular clustering (Figure 2A). Immunohistochemical staining for neurofilaments revealed a loss of myelin (Figure 2B). Within the lesion, astrocytes were reactive and exhibited polymorphic nuclei and prominent nucleoli (Figure 2C). After the presumptive diagnosis of PML, it was decided to continue treatment with mefloquine (250 mg/week) and dexamethasone (4 mg/day) for 6 months. Finally the PCR study of the biopsy specimen demonstrated the presence of JCV (5874 copies of JCV DNA /mL tissue suspension).

In the subsequent weeks, the neurological condition of the patient continued to deteriorate, with continuous epileptic fits, motor impairment with left hemiparesia, along with an increment in the extension and intensity of the parietal lesion, as revealed by a further MRI one month after diagnosis (Figure 1B). Thereafter, neurological condition and the MRI findings progressively improved and a year after diagnosis the patient achieved an almost complete resolution of the lesion as indicated by MRI, despite persisting residual motor impairment and epilepsy (Figure 1C). The patient is alive 38 months after the diagnosis. CLL continues to be in CR.

## Discussion

The significant aspects of this case are the difficulty of reaching the correct diagnosis and the long-term survival of the patient after the diagnosis of PML, which is the longest reported to date.

Risk factors for developing PML in HIV-infected people include low CD4+ lymphocyte count, whereas those in HIV-negative people remains a matter of debate [3]. In our patient, PML was associated with immunological dysfunction in relation to the baseline hematological disease, [7] receiving previous treatments (purine analogs, alkylating agents) [3] and/or monoclonal antibody therapy with rituximab [3,5]. All these elements, individually or in combination, led to an immunosuppression that has persisted in the patient to the present day, maintaining a low CD4+ count (339 cells/μL) 3 years after the treatment.

Establishing a diagnosis of PML requires the identification of JCV DNA in the CSF in conjunction with the characteristic neuroimaging findings or the demonstration of the typical histopathological triad (demyelination, bizarre astrocytes, and enlarged oligodendroglial nuclei), and coupled with the techniques to show the presence of JCV [8]. In the present case, the failure of PCR to demonstrate the JCV in CSF prompted us to perform a brain biopsy to establish a diagnosis, once the initial possibility of HVE became inconsistent and PML was the most likely possibility.

Several agents have been used to treat PML, although these treatments have been based on limited data, and none has proven consistently effective against JCV. *In vitro* studies of various compounds with anti-JCV activities showed that only mefloquine, an antimalarial agent, has sufficiently high penetration into the CNS. Additional experiments demonstrated that mefloquine inhibits the viral infection rates of three JCV isolates: JCV(Mad1), JCV(Mad4), and JCV(M1/SVEDelta). This effect is exerted in three cell types: transformed human glial (SVG-A) cells, primary human fetal glial cells, and primary human astrocytes. Mefloquine was also shown to inhibit viral DNA replication [9] when quantitative PCR was used to determine the number of viral copies in cultured cells.

Steroid treatment is usually given in HIV-negative PML-immune reconstitution inflammatory syndrome (IRIS) patients in an effort to dampen the inflammatory response. Although some published cases showed possible benefits, [10] the use of steroids to treat PML remains controversial because these medications are immunosuppressants and may contribute to increase HIV replication in HIV-positive patients, or disrupt treatment plans in HIV-negative patients with cancer or autoimmune diseases.

The survival of PML in patients with a hematological malignancy is less than 3 months [3,7]. Carson reported an overall case-fatality rate of 90%: 100% among PML cases diagnosed within 3 months of the last rituximab dose versus 84% among PML cases diagnosed more than 3 months after the last rituximab dose [3].

In this large series of 57 cases of HIV-negative patients treated with rituximab only 5 patients survived: 2 received no therapy, 1 received cytarabine, another mirtazapine, and the last received cidofovir, donor lymphocyte infusions, cytarabine and risperidone. None of these patients received the mefloquine and dexamethasone combination.

Clifford et al., in a clinical research, could not demonstrate any concluding results about the benefits of mefloquine in the treatment of PML. The small number of evaluable patients in this trial reflects the logistical and technical challenges inherent in PML clinical studies and larger investigations are needed to reach definitive conclusions [11].

The present case is one of the few examples of long-term survival of this condition, although it is not possible to determine whether the treatment with mefloquine influenced this outcome.

### Consent

Written informed consent was obtained from the patient for publication of this Case report and any accompanying images. A copy of the written consent is available for review by the Editor-in-Chief of this journal.
